# Percutaneous Coronary Intervention on Right Coronary Artery With All Coronary Arteries From Three Separate Ostiums in the Right Sinus of Valsalva

**DOI:** 10.4021/cr29e

**Published:** 2011-07-25

**Authors:** Muhammet Rasit Sayin, Mustafa Aydin, Sait Mesut Dogan, Turgut Karabag

**Affiliations:** a Zonguldak Karaelmas University, School of Medicine, Department of Cardiology, Kozlu 67600, Zonguldak, Turkey

**Keywords:** Coronary artery anomalies, Percutaneous coronary intervention

## Abstract

Some of coronary artery anomalies, such as origin of all coronary arteries from three separate ostiums in the right sinus of valsalva, represent a small amount of coronary anomalies. We describe a 63-year-old female patient which coronary angiogram revealed an origin of all coronary arteries from three separate ostiums in the right sinus of valsalva, with significant atherosclerotic plaque at the midportion of the right coronary artery. The stenosis was treated through percutaneous coronary intervention.

## Introduction

Coronary artery anomalies are well known clinical entities. The incidence of coronary artery anomalies has been reported between 0.6% and 1.6% in different angiographic series [1, 2]. Some of these anomalies, such as origin of all coronary arteries from three separate ostiums in the right sinus of valsalva (RSV), represent a small fraction of coronary anomalies. We describe a 63-year-old female patient with acute myocardial infarction (MI) treated with thrombolytic therapy. Because of resting angina, coronary angiography was performed. The coronary angiogram revealed an origin of all coronary arteries from three separate ostiums in the RSV, with significant atherosclerotic plaque at the midportion of the right coronary artery (RCA). The stenosis was treated through percutaneous coronary intervention (PCI).

## Case Report

A 63-year-old female was admitted with the complaint of typical chest pain suggesting MI. The electrocardiogram showed acute inferoposterolateral MI. She had a medical history of diabetes mellitus, hypercholesterolemia and premature coronary artery disease in her family, with no history of hypertension or smoking. Physical examination was normal. Laboratory data revealed raised troponin I 1.15 ng/mL (normal < 0.04 ng/mL) and mass creatine kinase-MB 32.3 ng/mL (normal < 6.3 ng/mL). Thrombolytic therapy was administered and ST segment resolution was occurred on electrocardiogram.

**Figure 1:**
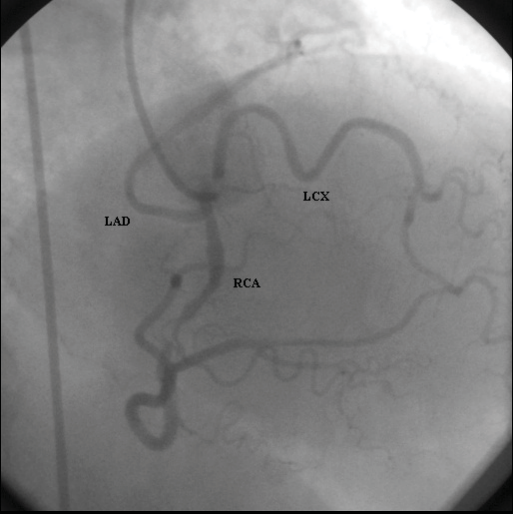
Coronary angiogram revealed all coronary arteries from three separate ostiums in the right sinus of valsalva.

During hospitalization angina was recurred and invasive strategy was considered. Coronary angiography did not demonstrate any vessel originating from the left sinus of valsalva. However, originating from the RCA, left coronary artery (LAD) and left circumflex artey (LCX) on the condition of having separate ostiums from the RSV was observed (fig. 1).

**Figure 2:**
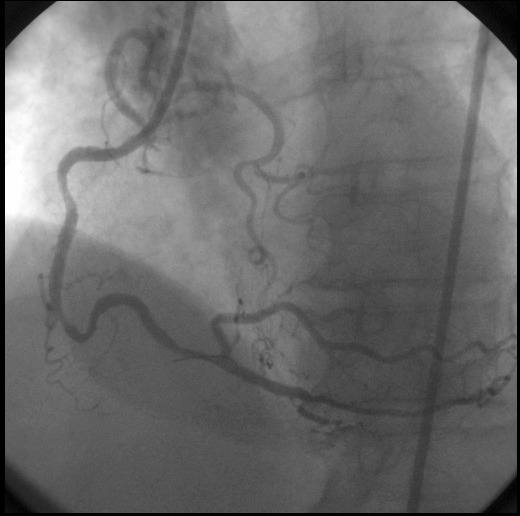
Post stenting coronary angiogram.

The LAD and LCX arteriogram showed no significant atherosclerotic disease. In the midportion of the RCA a significant atherosclerotic plaque was demonstrated. The RCA was selectively cannulated using a 7 F Medtronic Launcher right guiding catheter. The lesion was crossed with a 0.014 inch 180 cm floppy J (Boston Scientific Int.) guidewire and a 3.0 × 17 mm Presillion (Cordis Corporation, Florida, USA) stent was primarily placed without predilatation (fig. 2). No complication was occurred.

The patient was discharged 4 days later without symptoms. Beta blocker, angiotensin converting enzyme inhibitor, clopidogrel, atorvastatin and acetylsalicylic acid were prescribed. During 4 months outdoor follow-ups the patient had no trouble.

## Discussion

The existing case in literature that the all coronary arteries originating from the RSV with separate ostiums is rare. To the best of our knowledge, this is the first case report in the literature describing PCI in patients with all coronary arteries from three separate ostiums in the RSV with the culprit lesion being the RCA [3, 4]. Although it is generally in a benign character, some part of coronary artery anomalies constitutes a malignant clinical view like congestive heart failure, arrhythmia, myocardial infarction, syncope, and sudden deaths [5]. Some patients with this anomaly have symptoms of angina pectoris due to atherosclerosis of the coronary arteries as was the case in our patient. In our patient the LAD and LCX arteriogram showed no significant atherosclerotic disease while the severe stenosis of RCA was treated with direct stenting with a bare metal stent. The therapeutical choice depends on the site and character of the lesion. All cardiologists should be aware of anatomical variations in coronary circulation for making accurate diagnosis and selecting best treatment option.
